# Effect of Die Channel Geometry on the Quality of Pellets Obtained from Biomass

**DOI:** 10.3390/ma19010079

**Published:** 2025-12-25

**Authors:** Jakub Styks, Marek Wróbel

**Affiliations:** Department of Mechanical Engineering and Agrophysics, University of Agriculture in Krakow, Al Mickiewicza 120, 31-120 Kraków, Poland

**Keywords:** biomass, densification, pellets, geometry of compaction channel, geometry, pressure

## Abstract

The presented research focuses on assessing the impact of the geometry of the compaction channel on the quality of pellets produced from giant miscanthus, silphium, and sida. Geometry refers to parameters such as *L*, *D*, α, and the diameter of the channel, as well the height of the compacting cone. Our analysis covered the pressure compaction process of monocotyledonous and dicotyledonous perennials, considered a valuable source of biomass for energy purposes. These species are the subject of processing research; they are promising, easy to grow and, crucially, non-invasive. The results of the research indicated the optimal configurations for each plant. For miscanthus: *D* = 12 mm, α = 10°, *L* = 13 mm, and compaction pressure *P* = 245 MPa; for *D* = 10 mm, *α* = 10°, *L* = 22 mm, and *P* = 185 MPa. For silphium: *D* = 12 mm, *α* = 20°, *L* = 21 mm, *P* = 50 MPa, and for *D* = 10 mm: α = 20°, *L* = 26 - 27 mm, and *P* = 42 MPa. For Virginia mallow: *D* = 12 mm, *α* = 10°, *L* = 5 mm, and *P* = 237 MPa, or with a diameter of 10 mm: *α =* 30°, *L* = 23 mm, and *P* = 58 MPa.

## 1. Introduction

In order to meet the increasingly stringent requirements imposed on the global energy sector, solutions are being sought that will enable the production of environmentally friendly fuel. One area of research is the possibility of producing compacted solid biofuels from biomass. The aim is to obtain fuel with uniform quality characteristics from a wide variety of materials—biomass from different plant species.

### 1.1. Pressure Densification—Process Background

The process involving the pressure densification of materials is widely known and used in many branches of industry [1,2,3,4]. This is a process that is continually being refined [5], because not only does pressure determine its course, but a whole range of other factors also significantly influence the parameters of the obtained granules, compacts, tablets, pellets, or briquettes. While pressure is the primary driver of the process, the remaining variables can be categorized into three distinct groups:

- Material parameters—moisture content, particle size distribution, chemical and structural composition [6], specific density, and bulk density of the raw material;
- Technological parameters—specific heat level required for the process, compaction speed, and time;
- Structural parameters [7]—chamber geometry.

The presence of this process in many branches of industry confirms its universality, but also indicates how important this process is. The main essence of the compaction process is the reduction in free intermolecular spaces in the compacted material, and consequently, the increase in the contact surface between particles, enabling the formation of permanent bonds between them. Consequently, it becomes possible to produce granules possessing a precise geometrical form. The densification achieved during compaction arises from three primary mechanisms: the deformation of particles, their fragmentation (crushing), and the collapse of internal pore structures within the particles. The dominant mechanism varies with the material type [8].

Pressure compaction is a process that, due to its characteristics, is beneficial from a logistical perspective. It facilitates storage, reduces storage costs, and lowers the cost of transporting the granulate. Another advantage is the uniform raw material composition of the mixture and ultimately of the final product [9,10]. This process allows us to maintain a uniform, desired shape [11]. In the case of bulk materials, the appropriate parameters of the compaction process allow for a reduction in dust generation during material processing.

This method is extensively employed in the conversion of biomass for energy production [12,13,14,15]. As a result, biomass subjected to compaction takes on the two most well-known forms of solid biofuels, namely briquettes and pellets. This process is carried out using technical systems—from separate workstations to large production lines—which operate in similar ways and can be conducted in an open or closed chamber. Compaction in a closed chamber occurs in several stages.

The first stage is material preparation; in the case of biomass, the material is seasoned, dried, and shredded to an appropriate particle size. At this stage, it is also possible to mix the base material with a binder. The next stage is loading the raw material into the closed (pressing) chamber. This chamber is sealed to prevent material loss and to ensure controlled process conditions. Then, the material is compacted under pressure, where piston displacement results in volume reduction in the material. Friction occurring during the process generates heat, causing natural binders like lignin or resins to plasticize. This plasticity allows for the formation of stronger intermolecular bonds. After achieving the desired compaction pressure, the chamber is opened, and the finished product in the form of a briquette is ejected using the piston. In this case, the geometry of the compaction chamber determines both the shape and density of the product [16]. In this type of process, the pressure should be properly adjusted to the compacted material so that the final product meets the specified quality criteria.

Pressure compaction can also be carried out using technical systems with an open compaction chamber. The sleeve serving as the chamber has a narrowing outlet channel that is responsible for the compaction [17,18,19]. Due to the occurrence of external friction, the material encounters increasing resistance when moving through the compaction sleeve, resulting in its gradual compaction. In this case, the decisive factors are the individual characteristics of the material as well as the geometry of the sleeve [17,18,19,20,21].

### 1.2. Role of Die Geometry in Process

In pellet production lines, a slightly different solution of an open compaction chamber is used. Such a chamber consists of a compaction cone with an entry angle characteristic of a given compacted material, and a compaction channel with appropriate diameter and length. The material located on the surface of the die is forced into the compaction cone, whose task is to preliminarily compact the material and introduce it into the compaction channel. In the compaction channel, due to the friction and pressure generated by each subsequent portion of injected material, the material is compacted into its final form, i.e., a pellet [14,22,23,24]. When using this type of technical system, it is crucial to properly design the geometry of both the conical and cylindrical sections, because appropriate selection will impact the energy consumption of the process, as well as the quality of the resulting product. During the selection process, material parameters must be taken into account, since materials of even seemingly similar origin may require the use of different process parameters.

Taking the above into account, it should be noted that the process of pressure compaction of biomass depends largely on and is adapted to the material that will undergo this process. As mentioned, a large part of published research focuses on processes carried out on stands with a closed chamber [5,12,13,25,26,27], and based on these, it is not possible to unambiguously determine the optimal geometry of the compaction channel.

Available sources indicate that research is being conducted on compaction using stands designed to simulate the actual compaction process, but it should be noted that there are few such studies [1,24,28]. This type of research provides important data, but conducting it properly is more complicated because the material to be compacted can flow freely through the compaction chamber, and no element assists in retaining it [22,29]. The most important issue addressed in these studies is the proper selection of compaction die parameters to match the characteristics of the agglomerated material, since these parameters are responsible for retaining the material in the compaction chamber and its compacting [30,31].

In the literature addressing the topic of pressure compaction, a number of research studies have been undertaken aimed at determining the influence of the compaction chamber geometry, particularly the cone divergence angle, on the parameters of biomass granulation process.

According to study [23], where the subject of research was woody biomass, the energy consumption of the process using a 60° cone was evaluated. It was found that for beech, this indicator reached 74%, while for pine, an increase of 66% was recorded compared to the reference process carried out in a cylindrical sleeve. In another approach, Križan [32] analyzed the relationship between the quality of the obtained granulate and the divergence angle (1°, 4°, 6°, 9°, 11°) for woody biomass. The study identified the optimal chamber geometry, defining a key parameter: the ratio of the material’s contact surface area to the chamber volume. It was observed that this parameter decreased proportionally to the increase in the angle value. Winter et al. [33], using waste material (RDF—Refuse-Derived Fuel), studied the influence of angles 0, 4, 14, and 28° on the efficiency of pellet production. The results indicated that the configuration with a 28° angle generated the highest pellet quality with simultaneously the lowest recorded compaction pressure. Mišljenović’s team [34] conducted experiments on wheat straw compaction, using both a single stand and a flat die with cones at a 37.6° angle. It was proven that the variant using a single stand resulted in the production of granules with higher mechanical strength (*DU*). Hu and his team [35] focused on measuring the energy consumption of the compaction process for rice grass, testing five angle values in the range of α = 29.5–60.5°. A wider range of angles (α = 15–120°) was considered in the research by Wu et al. [36], concluding that reducing the angle α resulted in a decrease in compaction force, which directly corresponded to a reduction in external friction.

A key geometric parameter of every pellet mill die is the compression ratio, expressed as the quotient of the compaction channel length (*L*) to its diameter (*D*), with values typically ranging from 4.0 to 7.5. Its optimal value is strongly dependent on the physicochemical characteristics of the input material, resulting from the diversity of raw material properties [37]. An increase in channel length (*L*) generates higher internal pressure values during compaction. The opposite relationship is observed for diameter (*D*)—its reduction decreases the force necessary to push the material through the channel. Therefore, the geometric parameters of the channel are considered decisive for the efficiency and stability of the pressure compaction technological process [38,39]. The analysis of the structure in which a given process takes place is an important issue that is widely researched and applied in the industry [40,41].

The influence of die channel configuration on the mechanical properties of the granulate has been the subject of numerous analyses. Heffiner and Pfost [42] experimentally confirmed that pellets produced using dies with minimal thickness, and thus the shortest compaction channels, exhibited the highest mechanical strength. Butler and McColly [43] reported that while maintaining constant process parameters (pressure, dose), the use of compaction chambers with smaller volumes resulted in granules with higher densities and larger dimensions. Tumuluru et al. [44], analyzing biomass granulation, demonstrated that the mechanical durability (*DU*) of pellets produced in a die with a hole diameter of 6.4 mm was significantly higher compared to pellets obtained in a 7.2 mm die. Additional confirmation of the influence of this parameter was provided by the research of Hill and Pulkkinen [45] on alfalfa, where maximum granulate durability was recorded for dies with a compression ratio of 8–10.

In summarizing the biomass compaction process, which is crucial for producing eco-friendly fuel, it is based on a reduction in free intermolecular spaces and increasing particle contact, which allows for the formation of durable granules with uniform shape.

The process of the pressure compaction of biomass into pellet form requires careful adjustment of the die parameters to material specifications. The optimization of die geometry affects not only process efficiency but also fuel quality, which is particularly significant in the context of growing environmental requirements in the energy sector.

### 1.3. Compaction Channel Geometry—Gaps in Knowledge

The examples mentioned above only partially demonstrate how essential it is to select appropriate process parameters, material characteristics, and compaction channel geometry to achieve a high-quality product with the lowest possible pressure accompanying the production process. As can be observed, these studies are conducted on various, often proprietary test stands, and the obtained results are difficult to compare.

A proprietary test stand was constructed to study the impact of the aforementioned parameters on the compaction process. This apparatus simulates the progression of compaction within an open chamber and provides the capability to modify essential geometric parameters—namely, channel length, entry angle, and inlet orifice diameter. A comprehensive description is provided in the authors’ related work [24].

This research aimed to determine the effect of channel geometry on pellet quality—specifically density and mechanical strength—and the associated compaction pressures.

## 2. Materials and Methods

### 2.1. Material

The research material was obtained from an energy crop plantation located at the Faculty of Production and Power Engineering of the Hugo Kołłątaj University of Agriculture in Krakow. The subject of the research comprises selected perennial species, representing both monocotyledonous plants (*Miscanthus* × *giganteus* Greef et Deu.), and dicotyledonous plants (*Sida hermaphrodita* (L.) Rusby, *Silphium perfoliatum* L.). They exhibit high raw material potential in the context of energy use, which is confirmed in the subject literature [46]. Therefore, these species are the subject of advanced plantation and scientific research, focusing on optimizing methods for converting biomass obtained from them.

A key advantage of the analyzed plants, particularly in the case of taxa of foreign origin, is the lack of invasive species status. This characteristic allows for controlled commercial cultivation with minimized risk of unwanted spread in natural ecosystems, which stands in clear contrast to the ecological behavior of expansive species such as *Reynoutria* sp. or *Solidago* sp.

The above characteristics of the research material mean that, starting from cultivation with a negligible impact on the environment, the biomass obtained is a valuable energy resource, and the biofuel produced from it will enable sustainable energy production. We can conclude that the need to pay attention not only to the development of the process, technology, or product, but also to the life-cycle assessment and sustainable production systems is currently widely established [47].

Scientific reports indicate that the quality parameters of biomass from the aforementioned species allow it to be classified as a promising input material for producing high-quality solid biofuels, particularly pellets and briquettes [26,48,49,50,51,52]. The relatively limited understanding of these taxa under industrial conditions creates an opportunity for the significant expansion of knowledge in this area. Moreover, the affiliation of the studied plants with distinct botanical families (Poaceae, Malvaceae, Asteraceae) implies significant diversity in the physicochemical properties of their biomass. This, in turn, necessitates the individual selection and precise control of technological parameters for their compaction process.

Prior to commencing the main research stages, the obtained shoots underwent a preparation process. All investigated parameters were determined using laboratory equipment based on standardized methods, methods recommended by laboratory equipment manufacturers, and to a large extent on proprietary methods developed for the purposes of this study.

To simplify the description of the research procedure, the full botanical nomenclature of the studied species—including English and Latin generic names and species epithets— has been abbreviated. Thus, in the subsequent parts of this work, the studied species will be referred to as miscanthus, silphium, and sida.

**Figure 1:**
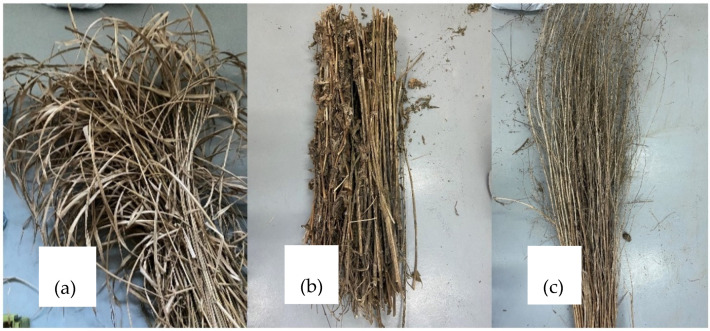
Research material collected from the plantation and prepared for seasoning: (**a**)—miscanthus; (**b**)—silphium; (**c**)—sida.

After harvesting, the shoots of the studied plants were bundled and left to season in a room with free air access. Bundles of the collected material are shown in Figure 1.

The seasoning process, involving natural drying in a room with free air access, continued until the material reached a moisture content below 10%. This moisture level halted the processes of biological material degradation and allowed for the proper execution of the next step, which was shredding the research material.

The total moisture content in the material was determined based on the procedure contained in the PN-EN ISO 18134-1:2023-02 standard [53].

### 2.2. Methods

#### 2.2.1. Grinding

The dried material was shredded to achieve a grain size d equal to or less than 1 mm. The grinding process was carried out in two stages. The first stage involved shredding the chopped material until the particle size did not exceed 6 mm. This was achieved using a knife mill with a 6 mm mesh screen (Testchem LMN-100, Radlin, Poland).

In the second stage, the raw material was shredded in a knife mill (PX-MFC 90D, Polymix, Kinematika, Luzern, Switzerland). The mill was equipped with a 1 mm mesh screen.

#### 2.2.2. Pressure Densification

The research plan assumed that the implementation of this stage should simulate the compaction process carried out in pellet machine dies as closely as possible. Therefore, unlike most studies on the biomass agglomeration process for energy purposes, it was assumed that material compaction would take place in an open chamber. To ensure proper densification of heating pellets, industry standards recommend constructing the die’s compaction chamber from two integrated sections: one cylindrical and one conical.

According to the definition contained in PN-EN ISO 16559, a pellet is a compacted biofuel produced from biomass with or without additives, taking the form of a cylinder with a maximum diameter of 25 mm and length up to 40 mm [54]. Depending on the raw material from which it is produced, it is divided into woody and non-woody pellets. In accordance with this classification, the requisite quality parameters are specified within the PN-EN ISO 17225-2 [55] and PN-EN ISO 17225-6 standard [56]. According to the quality guidelines for non-woody pellets (PN-EN ISO 17225-6), to be classified into quality classes A and B, in addition to meeting other requirements, they must have a diameter in the range of 6–25 mm. In the case of woody pellets (PN-EN ISO 17225-2), quality classes A1, A2, and B only permit diameters of 6 and 8 mm. Considering the above, the research assumed pellet production in an 8 mm diameter compaction channel. The cylindrical section length (*L*) was tested at five values: 5, 15, 25, 35, and 45 mm.

For the design of the conical section, dimensions were derived from a synthesis of literature review and die manufacturer specifications, it was assumed that the entry cone with apex angle α would be tested at 10, 20, 30, and 40°. Two research variants were adopted where the base diameter of the compaction cone, i.e., the entry diameter *D*, is 12 and 10 mm. In both cases, the cone truncation diameter equals the cylindrical part diameter and is 8 mm.

During the research, the compaction channel was heated to 100 °C, as this allows for the activation of the natural binders present in the material [57,58,59,60]. Based on the author’s previous work, it was determined that the material should be moisturized to 13% using a climate chamber [12,13].

**Figure 2:**
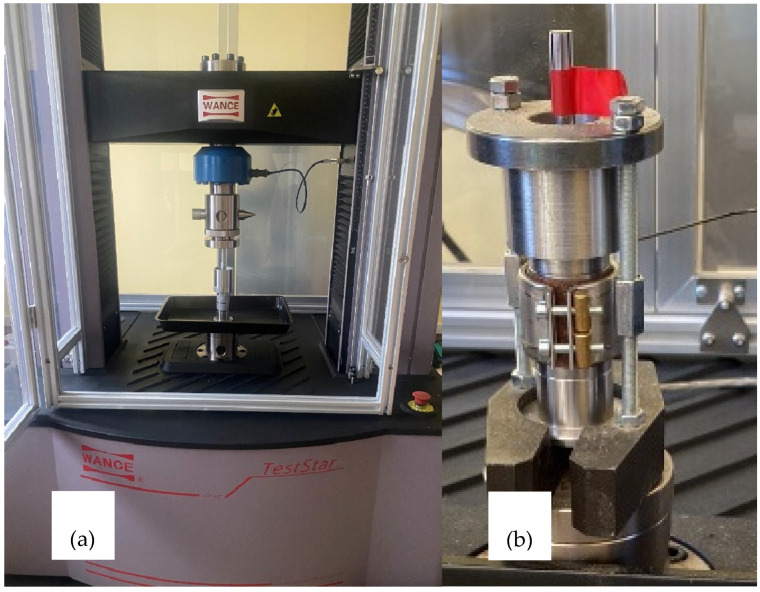
Research stand: (**a**)—Wance TestStar strength-testing machine; (**b**)—modular compaction attachment.

For each research variant (i.e., configuration of the aforementioned elements), the selected set of modules was assembled inside the housing. This assembly was subsequently mounted onto the grip of a testing machine (TestStar, Wance testing machine Co., Ltd., Shenzhen, China) (Figure 2). The piston displacement speed was 300 mm·min^−1^. The material dose compacted during a single piston stroke was 0.2 g; this mass was adopted based on [61]. The course of this research stage was described in detail in the author’s work [24].

During the test, the force variation relative to piston displacement was recorded. The compaction force for each experimental variant was defined as the recorded maximum force. Based on this, the pressure exerted on the surface of the compacted material (surface area of the entry cone base) was determined. Both the force and the exerted pressure are parameters that were not set during measurement but only recorded. The channel geometry, material, moisture content, and temperature determined what force would accompany the process. To rephrase, the maximum force output of each tested variant was measured.

For each research variant, 6–7 research pellets were produced. The produced pellets were placed in tightly sealed containers for 24 h, after which their mass, geometry, and quality parameters were determined, i.e., specific density *DE* and mechanical durability *DU* were used to quantify the impact of compaction channel geometry on pellet quality.

#### 2.2.3. Determination of Specific Density *DE* and Mechanical Durability *DU*

The obtained test pellets were evaluated in terms of quality parameters, i.e., *DU* and *DE*. While specific density was determined according to a standard testing procedure, the determination of mechanical durability required some modification of the standard testing method.

The specific density of the obtained research pellets was calculated based on the sample mass and its volume. A detailed description of the measurement procedure can be found in the author’s publications [12,13].

The mechanical durability of the research pellets was determined in a durability tester whose measurement chamber complied with the guidelines of the standard [62]. In the studied case, the mass of obtained granules for one measurement variant was significantly lower than required by the standard (500 g). Consequently, a modification was made to the test method: the sample mass prescribed by the standard was replaced by ballast material, i.e., 6 mm diameter balls made of ABS (acrylonitrile–butadiene–styrene terpolymer). The ballast mass was 950 g (this ballast mass occupied the same volume as a 500 g pellet sample).

As a result of these modifications, the mechanical durability of all obtained research pellets was determined according to the above method with the following parameters:

- Tester chamber compliant with ISO 17831-1:2025 [62];
- Chamber rotational speed—50 rpm;
- Ballast material—ø6 mm ABS plastic balls, mass 950 g;
- Number of rotations—740.

Considering that the average length of the test pellet used to verify the method was 17 mm, and that the mass of crushed material was similar regardless of the test pellet length, to eliminate the influence of pellet length on the *DU* result, the value of this parameter was determined from relation (1) with reference to a replacement pellet with a length *l_k_*_17_ of 17 mm (average length of test pellet ø8 mm adopted based on length measurement of pellets in a 1 kg sample):

(1) $${DU}_{17}\;=\;\frac{m_{A17}}{m_{E17}}\times 100$$

where

*DU*_17_—mechanical durability of the replacement pellet (%);

*m_A_*_17_—mass of the replacement pellet after the test (g);

*m_E_*_17_—mass of the replacement pellet before the test (g).

The mass of the replacement pellet *m_E_*_17_ was determined from the equation:

(2) $$m_{E17}\;=\;\frac{m_{E17}\times \;l_{k17}}{l_{k24}}$$

where

*m_E_*_17_—mass of the replacement pellet (g);

*l_k_*_24_—length of the test pellet (mm);

*m_E_*_24_—mass of the test pellet (g);

*l_k_*_17_—length of the replacement pellet (mm).

Whereas the mass of the replacement pellet after the test, *m_A_*_17_, which constituted the difference between the mass of the replacement granule *m_E_*_17_ and the mass of crushed material *m_R_*, was determined from the equation:

(3) $$m_{A17}\;=\;m_{E17}-\;m_{R}$$

where

*m_A_*_17_—mass of the replacement pellet after the test (g);

*m_E_*_17_—mass of the replacement pellet (g);

*m_R_*—mass of crushed material (g).

In turn, the mass of crushed material is the difference between the mass of the pellet before the test m_E24_ and the mass of the pellet after the test *m_A_*_:_

(4) $$m_{R}\;=\;m_{E24}-\;m_{A}$$

where

*m_R_*—mass of crushed material (g);

*m_E_*_24_—mass of the test pellet (g);

*m_A_*—mass of the test pellet after the test (g).

## 3. Results

### 3.1. Pressure

During compaction, the force necessary to extrude the material through the conical section and subsequently through the cylindrical module was recorded. The results were converted to pressure *P* to facilitate their comparison with other studies. The obtained relationships between compaction pressure and the tested variants are presented in the form of graphs.

#### 3.1.1. Miscanthus

**Figure 3:**
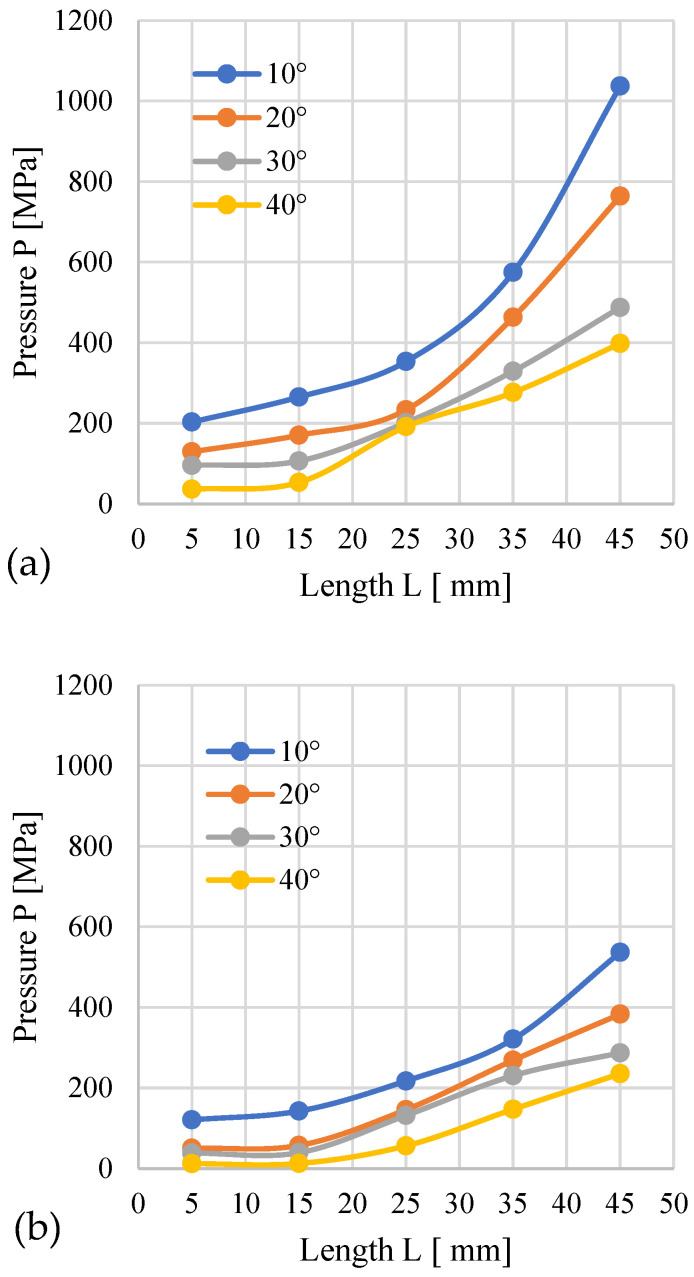
Effects of channel length (*L*), cone angle (*α*), and temperature on densification pressure (*P*) for miscanthus with 13% moisture: (**a**) size reduction: 12–8 mm; (**b**) size reduction: 10–8 mm.

Figure 3 shows the course of changes in the recorded compaction pressure (the pressure at which material displacement occurs in the die) depending on the length *L* of the cylindrical module of the die. In Figure 3a, the conical part has an entry diameter *D* = 12 mm, while in Figure 3b—*D* = 10 mm.

**Figure 4:**
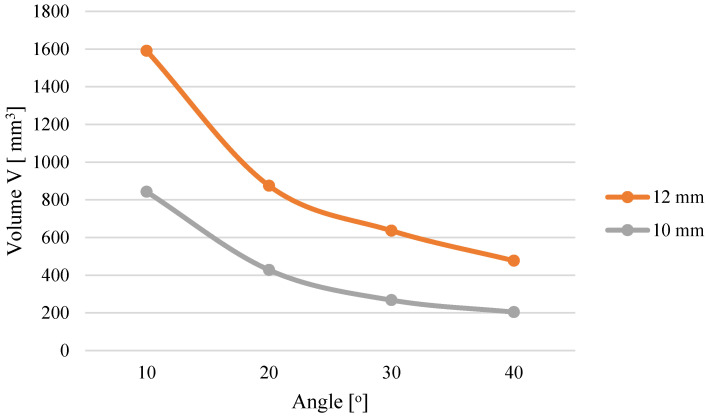
Effect of cone geometry on compaction cone volume [24].

The analysis indicates that higher pressure accompanies the process when the conical modules possess an entry diameter of *D* = 12 mm than with *D* = 10 mm. This influence applies to all tested materials. This results from the fact that regardless of the angle α, a cone with *D* = 12 mm has a larger volume compared to a cone with *D* = 10 mm (Figure 4). With the increased cone volume, due to the converging geometry of the cone, the contact surface area of the material expands, as presented in the author’s publication [24]. The cone also contains more material that needs to be pushed through the same *D* = 8 mm opening, which causes the pressure required to push the larger amount of material to be greater compared to a cone with smaller volume.

The increase in *L* causes an obvious increase in resistance and a rise in the pressure accompanying the process. For *D* = 12 mm, the highest pressure was recorded for α = 10° and *L* = 45 mm − 1037.5 MPa, and the lowest for α = 40° and *L* = 5 mm − 36.5 MPa (Figure 3a). Interestingly, with the decrease in angle α (for a given length *L*), an increase in pressure was observed. This effect is attributed to the increasing volume of the conical segment, which necessitates compacting a greater volume of material and subsequently forcing it into the 8 mm diameter cylindrical section. The volume increase is due to the fact that the base diameter of the conical section is constant at *D* = 12 mm. Exceeding the value of *L* = 25 mm causes a greater pressure buildup.

For modules with *D* = 10 mm, the pressure and its growth dynamics after exceeding *L* = 25 mm were lower. The highest pressure (536.61 MPa) was recorded for α = 10° and *L* = 45 mm, and the lowest (12.01 MPa) for α = 40° and *L* = 5 mm.

The obtained results confirm that both the module length *L* and the angle α influence the pressure recorded during the compaction of miscanthus biomass.

#### 3.1.2. Silphium

**Figure 5:**
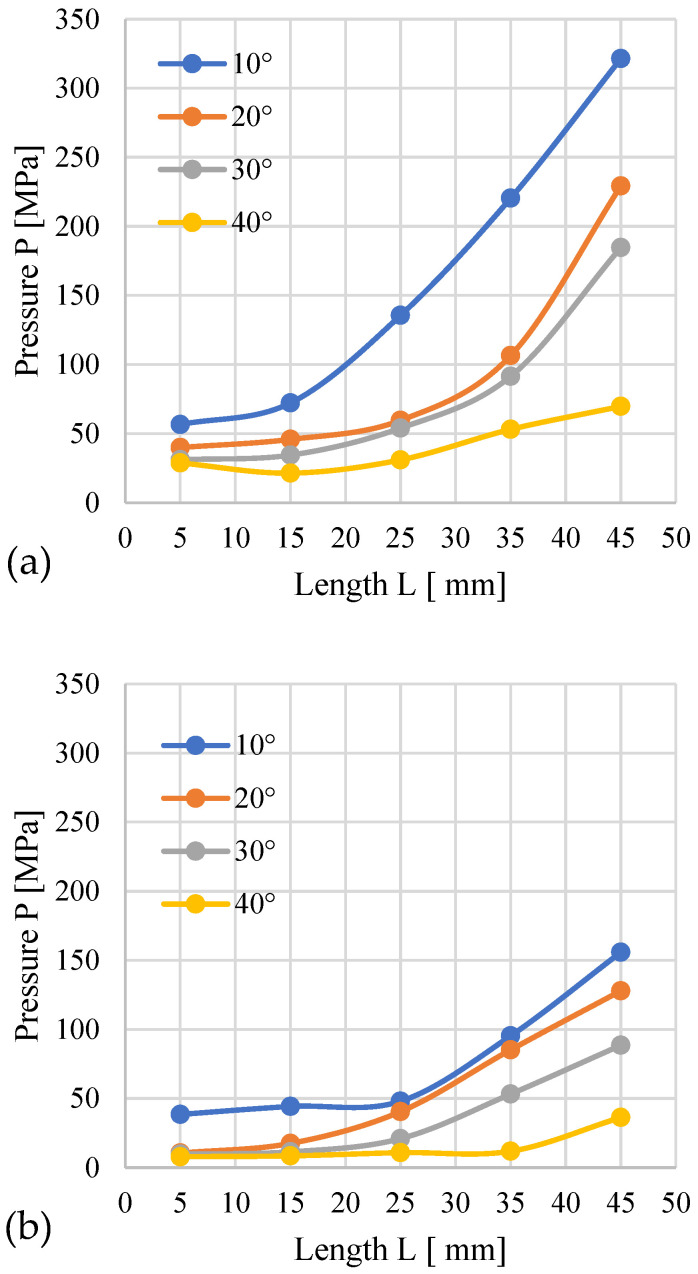
Effects of channel length (*L*), cone angle (*α*), and temperature on densification pressure (*P*) for silphium with 13% moisture: (**a**) size reduction: 12–8 mm; (**b**) size reduction: 10–8 mm.

The subsequent graphs (Figure 5) show the change in pressure depending on the geometry of the densification channel during the process of pelletizing silphium. Compared to miscanthus, the range of the recorded pressure is smaller.

For an inlet hole diameter of *D* = 12 mm, the highest pressure (321.43 MPa) was recorded for an angle of α = 10° and *L* = 45 mm, and the lowest (28.79 MPa) for α = 40° and *L* = 5 mm. The largest pressure difference, amounting to 251.67 MPa, was observed between the configurations α = 10°, *L* = 45 mm and α = 40°, *L* = 45 mm. It can also be noted that for α = 40°, no significant pressure changes occur across the entire range of *L*. In the case of α = 20° and 30°, a clear pressure increase occurs after exceeding *L* = 25 mm. For α = 10°, a clear pressure increase is already apparent after exceeding *L* = 15 mm.

For the 10 mm diameter channel, the pressure ranged from a minimum of 7.91 MPa (α = 40°, *L* = 5 mm) to a maximum of 155.88 MPa (α = 10°, *L* = 45 mm). Furthermore, a consistent pressure increase was observed for all tested α angles once the cylindrical length exceeded *L* = 25 mm.

**Figure 6:**
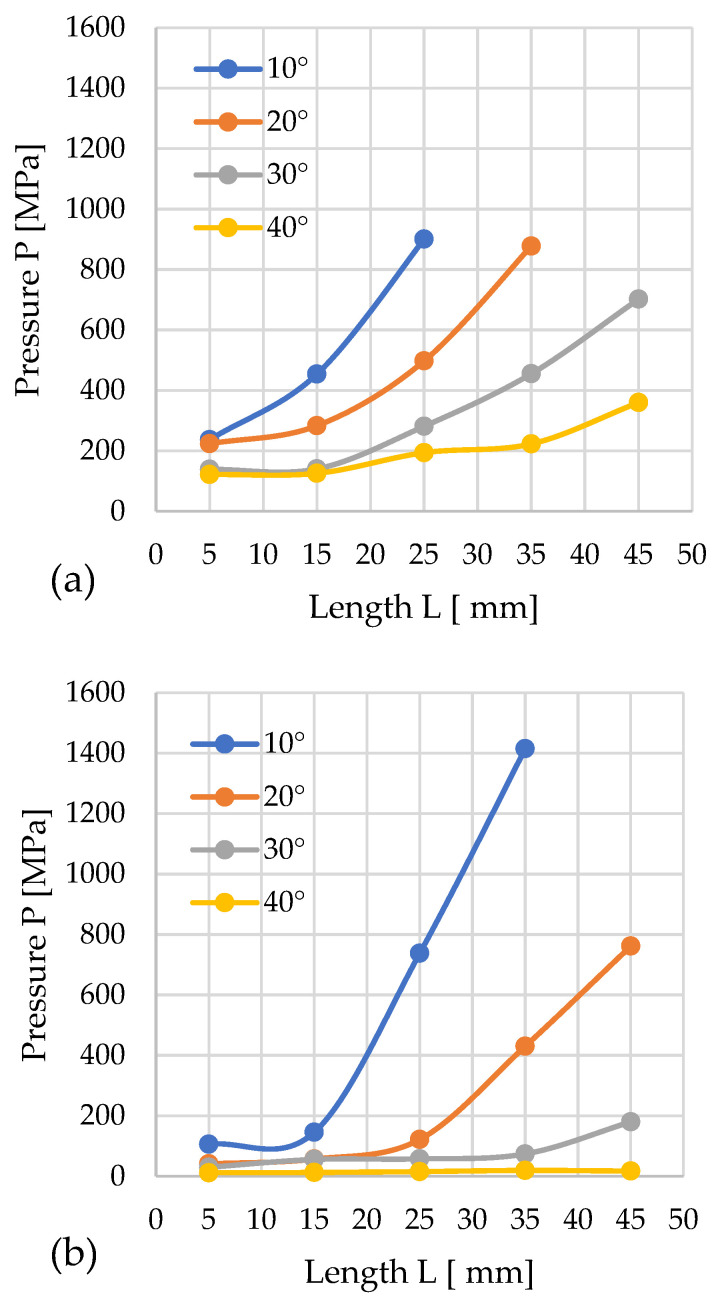
Effects of channel length (*L*), cone angle (*α*), and temperature on densification pressure (*P*) for 13% moisture sida: (**a**) size reduction: 12–8 mm; (**b**) size reduction: 10–8 mm.

The distinct pressure profiles observed for silphium and miscanthus demonstrate a significant material-dependent influence on the pressure densification process. This conclusion is further corroborated by the detailed pressure change profiles obtained for silphium (Figure 6).

#### 3.1.3. Sida

For sida, the minimum pressure in the 12 mm diameter channel was 122.4 MPa, which occurred with a cone angle of 40° and a cylindrical length (*L*) of 5 mm. In contrast, the highest pressure was noted for the configuration: *D* = 12 mm; *L* = 25 mm, α = 10°, *P* = 901.14 MPa.

For configurations with cones of diameter *D* = 10 mm, the lowest recorded pressure was 11.91 MPa for *L* = 5 mm, α = 40°. The highest result was obtained when compacting sida with the configuration *L* = 35 mm, α = 10°. The resulting pressure was 1414.7 MPa.

In several cases, as the α angle decreased, the process pressure increased sharply, reaching particularly high values for a cone angle of 10°, but also for α = 20°. The graphs show that the data series for *D* = 12 mm, α = 20°, and 10°, as well as for *D* = 10 mm and α = 10°, are incomplete. This is because the achieved pressure reached values exceeding the permissible range, which could damage the test stand, and did not result in material movement.

Analyzing the obtained results, it can be concluded that in the case of sida, this material is very sensitive to changes in *L* and α, except for the case where α = 40°. For α = 30° and *D* = 12 mm, the pressure increases after exceeding *L* = 15 mm, while for *D* = 10 mm, a slight increase was observed after exceeding *L* = 35 mm. The pressure curves have a different progression for α = 20°. For *D* = 12 mm, the pressure increases after exceeding *L* = 15 mm, and after exceeding *L* = 35 mm, the pressure increases beyond the measuring range of the stand. For *D* = 10 mm, the pressure begins to rise at *L* = 25 mm, reaching a value of 761.7 MPa at *L* = 45 mm.

As mentioned earlier, the graphs present only the pressure change profiles associated with the individual research variants. The pressure value is influenced by factors such as the system’s geometry and the type of material. Selecting a specific research variant allows for the pressure to be recorded, but its value is not directly regulated. It is also worth noting that the recorded pressure pertains only to the process of compacting the material up until the moment it begins to be displaced in the channel.

A key consideration involved determining if the tested setups could produce pellets satisfying mandatory quality metrics like specific density and mechanical durability. The subsequent step involved determining which configuration would induce the lowest pressures while simultaneously imparting the required values of the *DE* and *DU* quality parameters to the pellets.

From an economic perspective, the aim is to minimize process pressure; however, it is essential to achieve the required pellet density of *DE* ≥ 1 g/cm^3^ and a mechanical durability of *DU* ≥ 97.5%. Therefore, the optimal channel geometry allows us to achieve a suitable balance between minimal pressure and the required product quality.

### 3.2. Density

Among the critical quality parameters analyzed is the specific density (*DE*) of the pellets. The influence of the channel geometry on this parameter is presented in the graphs, using a layout analogous to the analysis of the pressure change profiles. It was assumed that the minimum specific density value meeting the quality criteria is 1 g/cm^3^. According to standard [63], class A1 pellets require a minimum bulk density of 600 kg/m^3^. While direct measurement of bulk density was precluded by the limited sample quantity, study [64] indicates that a bulk density of 600 kg/m^3^ corresponds to a specific density (*DE*) exceeding 1 g/cm^3^. This correlation was therefore adopted for assessment.

#### 3.2.1. Miscanthus

**Figure 7:**
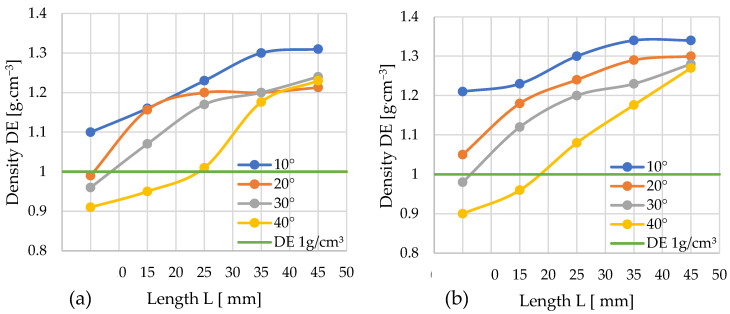
Effects of channel length (*L*), cone angle (*α*), and temperature on pellet density (*DE*) for miscanthus with 13% moisture: (**a**) size reduction: 12–8 mm; (**b**) size reduction: 10–8 mm.

The two graphs (Figure 7) present the progression of specific density (*DE*) values for the aforementioned experimental variants. A horizontal green line, denoting the threshold of 1 g/cm^3^, is included to facilitate the visual interpretation of the results.

Graph 7a depicts the specific density (*DE*) results from the densification process using the conical module with *D* = 12 mm. For the smallest cone angle (*α* = 10°), all applied cylindrical modules yielded pellets meeting the required density standard (*DE* > 1 g/cm^3^), with the maximum value of 1.31 g/cm^3^ recorded for the longest module (*L* = 45 mm). For *α* = 20°, the shortest module (*L* = 5 mm) failed to achieve the required density; the 1 g/cm^3^ threshold was only exceeded at *L* ≈ 7–8 mm. For this angle, maximum density was attained at *L* = 25 mm, with no significant further increase observed for longer cylindrical sections. A similar pattern was noted for *α* = 30°—only the shortest module (*L* = 5 mm) resulted in a substandard density, while the required value was achieved at *L* ≈ 9 mm. The steepest angle (*α* = 40°) enabled the production of normatively compliant pellets only when combined with a relatively long cylindrical module (*L* = 25 mm); the use of shorter modules resulted in insufficient density values.

By comparing the obtained profiles of *DE* change with the profiles of pressure *P* change, the minimum channel length *L* required to achieve the required *DE* threshold at the minimal pressure *P* can be determined for each studied angle α. For: α = 10°—*L*≈ 5 mm and *P* ≈ 204 MPa; α = 20°—*L* ≈ 7–8 mm and *P* ≈ 132 MPa; α = 30°—*L* ≈ 9 mm and *P* ≈ 105 MPa; and α = 40°—*L* ≈ 25 mm and *P* ≈ 190 MPa.

Four variants of the die channel geometry were obtained, which allowed us to achieve the required pellet density. However, the configuration with α = 30° and *L* ≈ 9 mm required the lowest compaction pressure (105 MPa) and, consequently, the lowest energy input.

Figure 7b presents the specific density (*DE*) data obtained using the conical module with *D* = 10 mm. For an angle of α = 10°, every tested configuration achieved a density *DE* > 1 g/cm^3^, irrespective of the cylindrical module length, with the highest recorded value of 1.34 g·cm^−3^ corresponding to the combination of *α* = 10° and *L* = 45 mm.

Similarly, for *α* = 20°, all configurations met the normative density requirement. In contrast, for *α* = 30°, the shortest cylindrical module (*L* = 5 mm) failed to produce pellets with the required density; the 1 g/cm^3^ threshold was only exceeded at a length of *L* = 6–7 mm.

The module with an angle α = 40° has the fewest combinations with cylindrical modules that achieve the target pellet density. For the *α* = 40° angle, configurations with cylindrical modules of *L* = 5 mm and *L* = 15 mm fail to meet the normative density requirements. Increasing the length of the cylindrical module positively impacts the densification process, as each subsequent combination from *L* = 25 mm to *L* = 45 mm yields a higher density, enabling the production of pellets with the desired specifications. Theoretically, the optimal module length for producing the highest quality pellets at *α* = 40° would be within the range of *L* = 18–19 mm.

A comparative analysis of the pellet density (*DE*) and pressure (*P*) change graphs allowed us to determine, for each angle α, the minimum channel length (*L*) necessary to achieve the required *DE* threshold while simultaneously minimizing pressure. For: α = 10°—*L* = 5 mm and *P* ≈ 121 MPa; α = 20°—*L* ≈ 5 mm and *P* ≈ 51 MPa; α = 30—*L* ≈ 6–7 mm and *P* ≈ 37 MPa; and α = 40°—*L* ≈ 18–19 mm and *P* ≈ 26 MPa.

Among the four available channel geometry variants satisfying the target pellet density condition, the configuration with an angle of α = 40° and a length of *L* ≈ 18–19 mm is characterized by the lowest compaction pressure (26 MPa). This directly translates to the minimization of energy input for the entire process.

#### 3.2.2. Silphium

**Figure 8:**
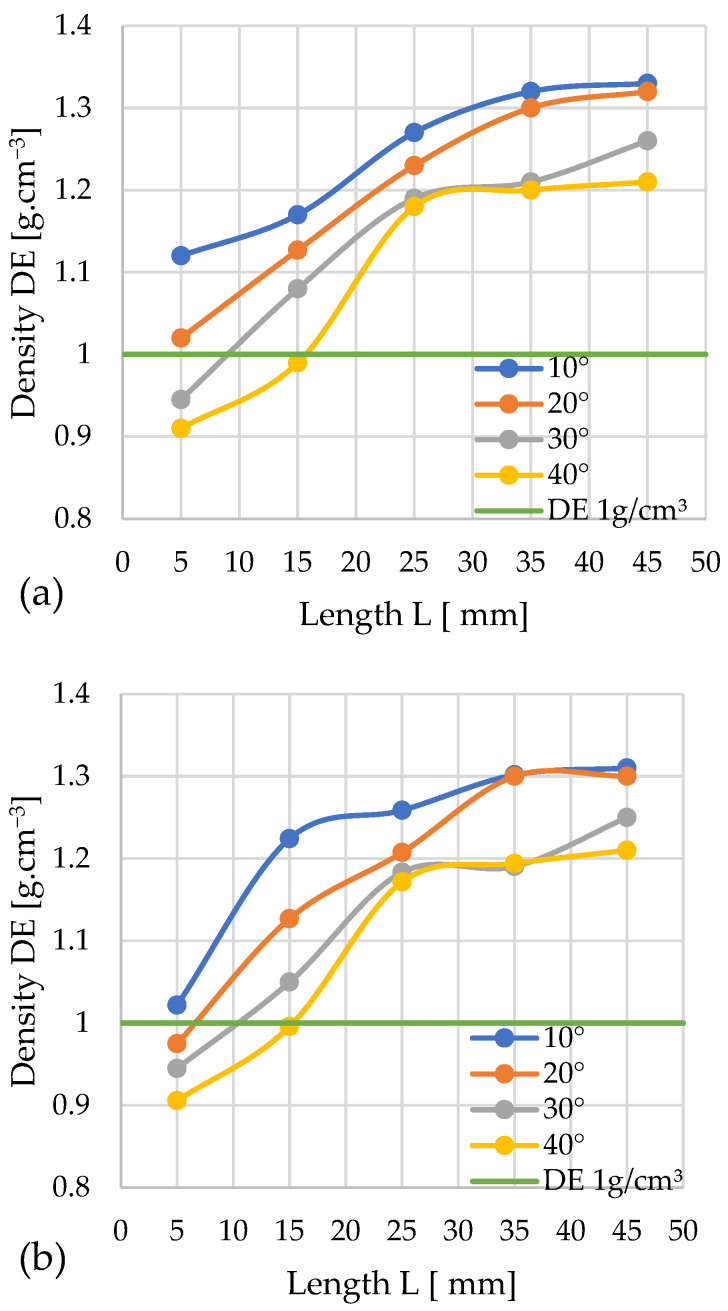
Effects of channel length (*L*), cone angle (*α*), and temperature on pellet density (*DE*) for silphium with 13% moisture: (**a**) size reduction: 12–8 mm; (**b**) size reduction: 10–8 mm.

The following graphs (Figure 8a,b) illustrate the progression of specific density (*DE*) changes as a function of the densification channel geometry for silphium biomass.

Using the *D* = 12 mm cones, just three configurations resulted in pellets below the required density (*DE* ≥ 1 g/cm^3^). These were the configurations: *L* = 5 mm, α = 30° and 40°, and *L* = 15 mm, α = 40°. The remaining variants met the quality criterion. The lowest density meeting the criteria was achieved with the configuration α = 20° and *L* = 5 mm, and the obtained pellet had a density of *DE* = 1.02 g/cm^3^. Pellets produced with the α = 10° and *L* = 45 mm achieved the highest level *DE* = 1.33 g/cm^3^. Thus, a simultaneous increase in pellet density with the lengthening of the densification channel can be observed.

A comparative analysis of the pellet density (*DE*) and pressure (*P*) profiles enabled the determination, for each angle α value, of the minimum channel length (*L*), ensuring the required *DE* threshold was achieved while simultaneously possessing a minimized pressure value. For: α = 10°—*L* = 5 mm and *P* ≈ 57 MPa; α = 20°—*L* = 5 mm and *P* ≈ 29 MPa; α = 30°—*L* ≈ 9 mm and *P* ≈ 32 MPa; α = 40°—*L* ≈ 16 mm and *P* ≈ 22 MPa.

Among the four available geometry variants satisfying the target pellet density condition, the configuration with an angle of α = 40° and a length of *L* ≈ 16 mm stands out for having the lowest compaction pressure (22 MPa).

Looking at the graph for *D* = 10 mm, one can observe an increase in the number of variants that do not guarantee obtaining pellets meeting the quality criteria. These configurations are characterized by the following parameters: α = 20°, 30°, 40°; *L* = 5 mm and α = 40°; *L* = 15 mm. All other configurations met the quality criteria.

The configuration yielding the lowest density that still met the quality standards for the produced pellets was α = 10°, *L* = 5 mm, with the pellet achieving a density of *DE* = 1.02 g/cm^3^. The highest pellet density was obtained using the following geometry: α = 10°, *L* = 45 mm, with a value of *DE* = 1.31 g/cm^3^.

A comparative analysis of the pellet density (*DE*) and pressure (*P*) profiles allowed us to determine, for each angle α value, the minimum channel length (*L*) enabling the achievement of the required *DE* threshold at minimized pressure. For: α = 10°—*L* = 5 mm and *P* ≈ 39 MPa; α = 20°—*L* ≈ 6–7 mm and *P* ≈ 11 MPa; α = 30°—*L* = 10 mm and *P* ≈ 11 MPa; α = 40°—*L* ≈ 15 mm and *P* ≈ 8 MPa.

Among the four identified channel geometry variants satisfying the target pellet density condition, the configuration with an angle of α = 40° and a length of *L* ≈ 15 mm is characterized by the significantly lowest compaction pressure (8 MPa). This parameter directly determines the energy intensity of the process, pointing to this configuration as the solution with the lowest energy input.

Once again, it can be observed that using a compression cone with an angle of α = 10° guarantees, in every combination, the production of a pellet achieving a density of *DE* ≥ 1 g/cm^3^, and the density obtained using this cone is always higher compared to other combinations. Similarly to the case of *D* = 12 mm, pellets produced using cones with an angle of α = 40° exhibited the lowest density relative to the other test series, with the exception of *D* = 10 mm, *L* = 35 mm.

The graphics also show that changing the inlet hole diameter *D* from 12 mm to 10 mm favorably influences the silphium densification process, as it significantly reduces the pressure accompanying the process, which is highly desirable from an economic standpoint.

#### 3.2.3. Sida

**Figure 9:**
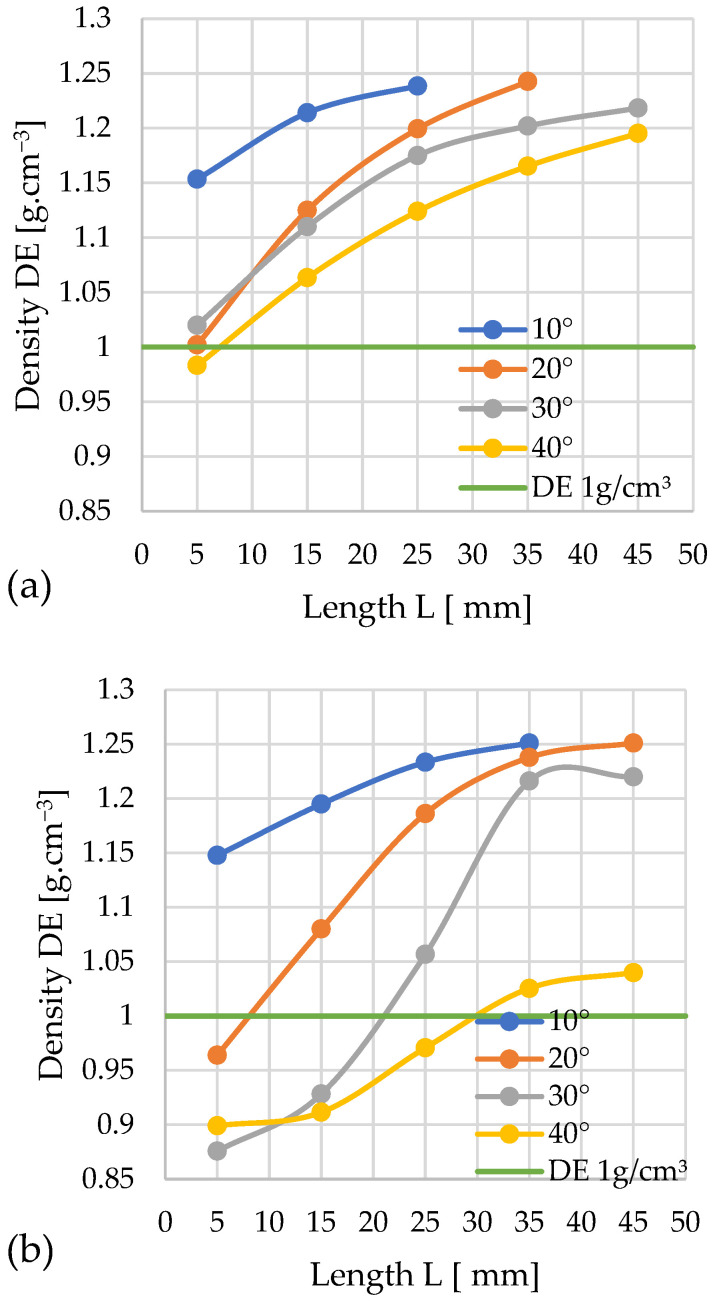
Effects of channel length (*L*), cone angle (*α*), and temperature on pellet density (*DE*) for sida with 13% moisture: (**a**) size reduction: 12–8 mm; (**b**) size reduction: 10–8 mm.

The pair of graphs (Figure 9a,b) shows the progression of changes in *DE* depending on the geometry of the densification channel during the compaction of sida.

The obtained results show that almost all variants meet the assumed quality threshold (for the cone diameter *D* = 12 mm). Only the pellet obtained with a cone angle of 40° and a compaction chamber length of 5 mm fell slightly below the threshold, with a density of 0.98 g/cm^3^. The highest recorded density of 1.24 g/cm^3^ was achieved by a pellet compacted with a 20° cone and a channel length of 35 mm.

By correlating the obtained relationships between *DE* and pressure change *P*, the minimum channel length *L* guaranteeing the achievement of the required *DE* threshold at the minimum pressure *P* can be determined for each studied angle α. For: α = 10°—*L* = 5 mm and *P* ≈ 237 MPa; α = 20°—*L* = 5 mm and *P* ≈ 224 MPa; α = 30°—*L* = 5 mm and *P* ≈ 139 MPa; α = 40°—*L* ≈ 7–8 mm and *P* ≈ 123 MPa.

This yields four variants of channel geometry that allow us to achieve the required pellet density. However, the configuration with α = 40° and *L* ≈ 7–8 mm requires the lowest compaction pressure (123 MPa), and consequently, the lowest energy input.

The results obtained for cones with *D* = 10 mm indicate that the best solution for compacting sida is a 10° angle, as using any compaction chamber length yields a pellet meeting the quality criterion. The highest density obtained in this test series, 1.25 g/cm^3^, was achieved by pellets compacted using both a 10° angle with a densification channel length of 35 mm and a 20° angle with a length of 45 mm. Conversely, the lowest result was obtained by compaction using a cone with a 30° angle and a channel length of 5 mm, achieving a density of 0.88 g/cm^3^. A 40° angle required a channel length exceeding 25 mm to achieve a density above the required threshold.

A comparative analysis of the *DE* and pressure *P* change profiles enables the determination, for each considered angle α, of the minimum channel length *L* guaranteeing the achievement of the required *DE* value threshold at the lowest pressure *P*. For α = 10°—*L* = 5 mm and *P* ≈ 106 MPa; α = 20°—*L* ≈ 8–9 mm and *P* ≈ 52 MPa; α = 30°—*L* = 22 mm and *P* ≈ 56 MPa; α = 40°—*L* = 30 mm and *P* ≈ 17 MPa.

Consequently, four variants of channel geometry enabling the achievement of the required pellet density were obtained. Among them, the most efficient solution, from the point of view of minimizing compaction pressure (17 MPa) and thus reducing the energy input of the process, is the configuration with an angle of α = 40° and a channel length of *L* = 30 mm.

### 3.3. Mechanical Durability

The mechanical durability (*DU*) of the pellets served as the second quality metric under investigation, with its dependence on channel geometry illustrated in the figures, analogously to the specific density (*DE*). Mechanical durability (*DU*) was determined for the same pellets that were previously subjected to *DE* measurement.

The maximum allowable mass loss of 2.5% was adopted as the quality criterion, which is consistent with the standard PN-EN ISO 17225-6:2021 for class A pellets and means *DU* ≥ 97.5% (the horizontal line represents the threshold *DU* value). The results were averaged and presented graphically, and data falling outside the repeatability range specified in the standard were omitted.

#### 3.3.1. Miscanthus

**Figure 10:**
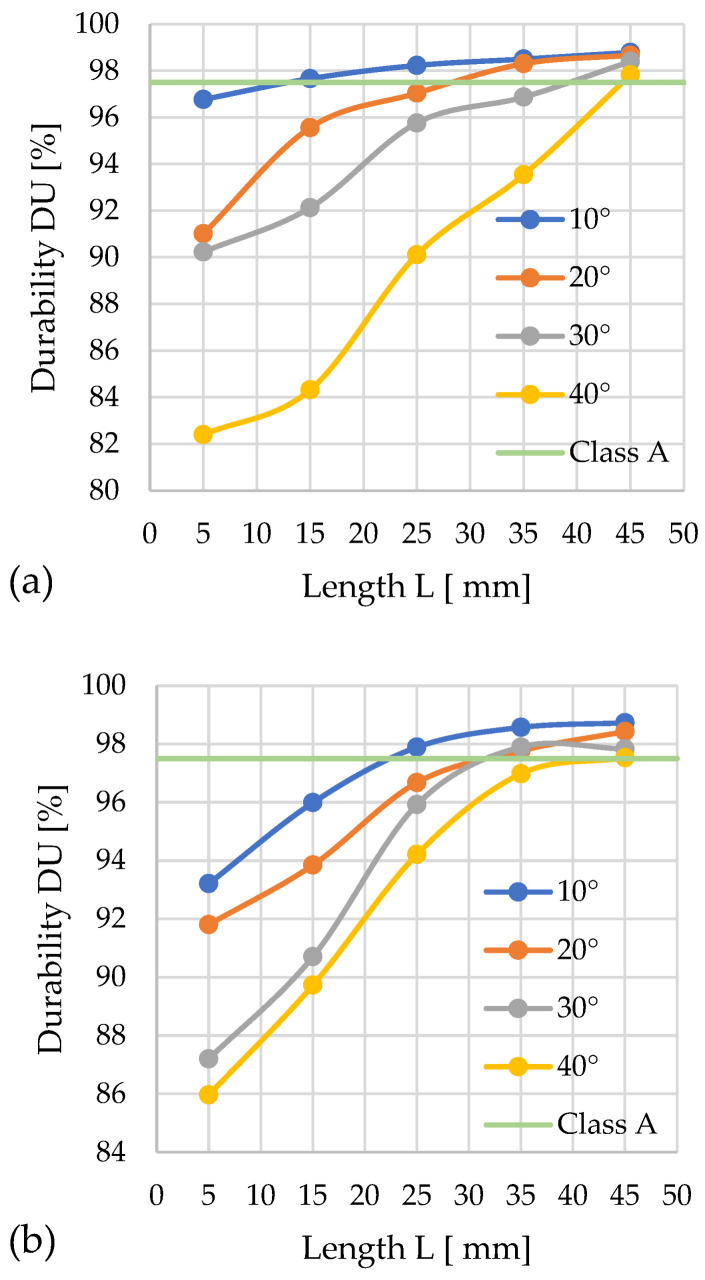
Effects of channel length (*L*), cone angle (*α*), and temperature on pellet durability (*DU*) for miscanthus with 13% moisture: (**a**) size reduction: 12–8 mm; (**b**) size reduction: 10–8 mm.

The complete set of graphs (Figure 10a,b) presents the results obtained for the change in mechanical durability of miscanthus pellets as determined by the geometry of the densification channel.

In the first graph (for *D* = 12 mm), it was observed that among the tested variants, eight qualified for class A, namely the configurations α = 10° with *L* = 15; 25; 35; 45 mm, α = 20° with *L* = 35; 45 mm, α = 30° with *L* = 45 mm, and α = 40° with *L* = 45 mm. The best result, *DU* = 98.8%, was achieved with a cone angle of α = 10° and *L* = 45 mm.

In the second graph (for *D* = 10 mm), eight variants also meet class A *DU* requirements: α = 10° with *L* = 25; 35; 45 mm, α = 20° with *L* = 35; 45 mm, α = 30° with *L* = 35; 45 mm, and α = 40° with *L* = 45 mm. The highest durability, *DU* = 98.7%, was achieved by pellets produced in the configuration α = 10° and *L* = 45 mm.

From the graphical comparison, it is evident that the key factors affecting pellet quality are the geometries of the densification channel and the cone.

The best parameters for the pressure densification of miscanthus for quality class A with diameter *D* = 12 mm are α = 10°, *L* = 13 mm, while for diameter *D* = 10 mm and quality class A, they are α = 10°, *L* = 23 mm.

Data analysis revealed the possibility of determining the minimum channel length *L* for each angle α value based on a comparison of the *DU* and pressure *P* change curves, ensuring the achievement of the required *DU* threshold for quality class A at minimized pressure values.

In the case of *D* = 12 mm: α = 10°—*L* = 13 mm and *P* ≈ 245 MPa; α = 20°—*L* ≈ 27 mm and *P* ≈ 276 MPa; α = 30°—*L* = 39 mm and *P* ≈ 375 MPa; α = 40°—*L* = 43–44 mm and *P* ≈ 357 MPa.

In the case of *D* = 10 mm: α = 10°—*L* = 22 mm and *P* ≈ 185 MPa; α = 20°—*L* ≈ 31–32 mm and *P* ≈ 211 MPa; α = 30°—*L* = 31–32 mm and *P* ≈ 200 MPa; α = 40°—*L* = 45 mm and *P* ≈ 235 MPa.

Among the possible geometric variants satisfying class A requirements, the configuration characterized by the lowest compaction pressure (185 MPa) and minimal energy input corresponds to the parameters: *D* = 10 mm, α = 10°, *L* = 22 mm.

#### 3.3.2. Silphium

**Figure 11:**
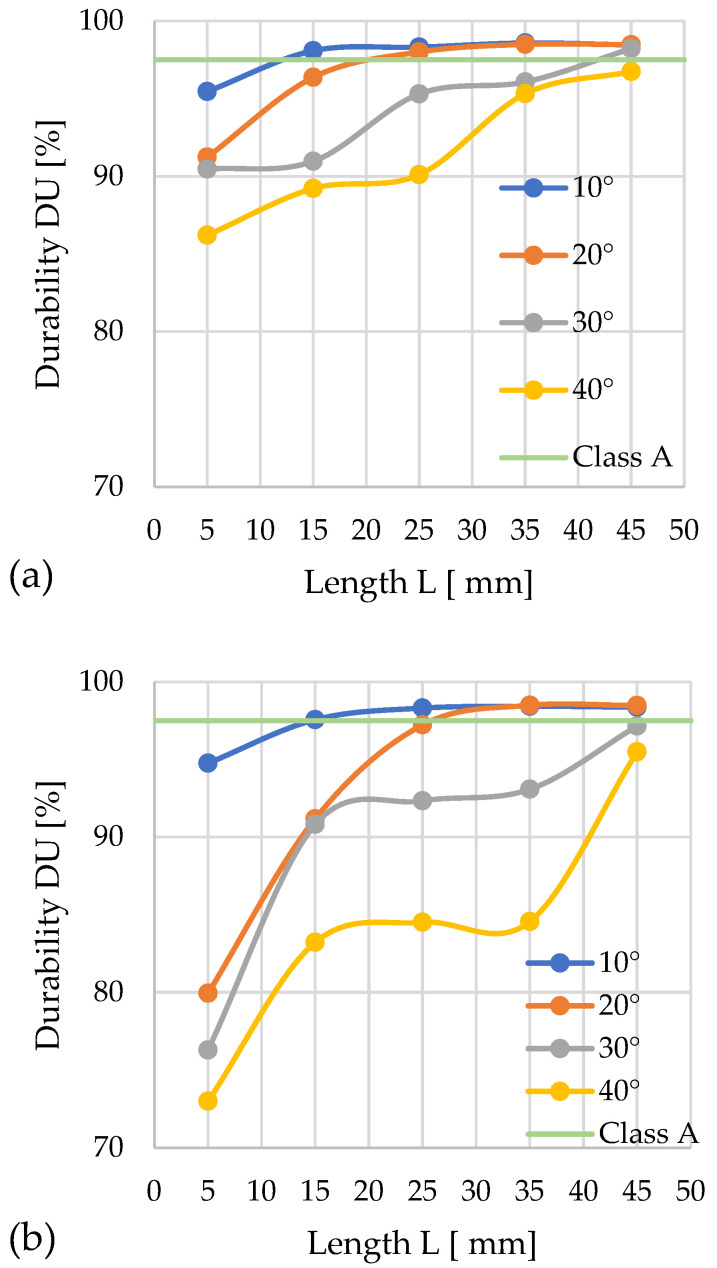
Effects of channel length (*L*), cone angle (*α*), and temperature on pellet durability (*DU*) for silphium with 13% moisture: (**a**) size reduction: 12–8 mm; (**b**) size reduction: 10–8 mm.

The following graphs show the progression of changes in *DU* (Figure 11a,b) in relation to the variable parameters of the densification channel.

For *D* = 12 mm, the lowest mechanical durability (*DU* = 86.2%) was achieved by pellets compacted with a cone angle of α = 40° and *L* = 5 mm. Among the analyzed configurations, class A was achieved by pellets produced in the following variants: α = 10° with *L* = 15, 25, 35, 45 mm; α = 20° with *L* = 25, 35, 45 mm; and α = 30° with *L* = 45 mm. The highest *DU* durability was observed for channel lengths of 35 mm, while extending them by 10 mm caused a slight decrease in this value.

For *D* = 10 mm, class A was achieved in the systems: α = 10° with *L* = 15, 25, 35, 45 mm; and α = 20° with *L* = 35, 45 mm. The greatest mechanical durability (*DU* = 98.5%) was obtained for α = 10° and *L* = 35 mm. Throughout the test series, the configuration with the α = 10° angle stood out for its high durability. The other configurations achieved low durability at channel lengths of *L* = 5 and 15 mm. The weakest results were recorded for α = 40°, where no channel length provided the required *DU* value.

A comparison of both inlet hole diameter variants indicates that the larger diameter (*D* = 12 mm) favors better compaction quality of silphium. With the larger diameter, more pellet groups met the quality requirements, and the range of obtained values was more stable.

Based on a comparative analysis of the *DU* parameter and pressure *P* change profiles, it was possible to establish for each angle α value the minimum channel length *L*, ensuring the achievement of the required *DU* threshold for quality class A at minimized process pressure.

In the case of *D* = 12 mm: α = 10°—*L* = 13 mm and *P* ≈ 65 MPa; α = 20°—*L* ≈ 21 mm and *P* ≈ 50 MPa; α = 30°—*L* = 42–43 mm and *P* ≈ 165 MPa; α = 40°—none of the configurations met the normative requirements.

In the case of *D* = 10 mm: α = 10°—*L* = 15 mm and *P* ≈ 44 MPa; α = 20°—*L* ≈ 26–27 mm and *P* ≈ 42 MPa; α = 30° and α = 40°—none of the configurations met the normative requirements.

Among the possible geometric variants satisfying class A requirements, the configuration characterized by parameters *D* = 10 mm, α = 20°, and *L* = 26–27 mm provided the lowest compaction pressure (42 MPa) and simultaneously minimal energy input for the process.

#### 3.3.3. Sida

**Figure 12:**
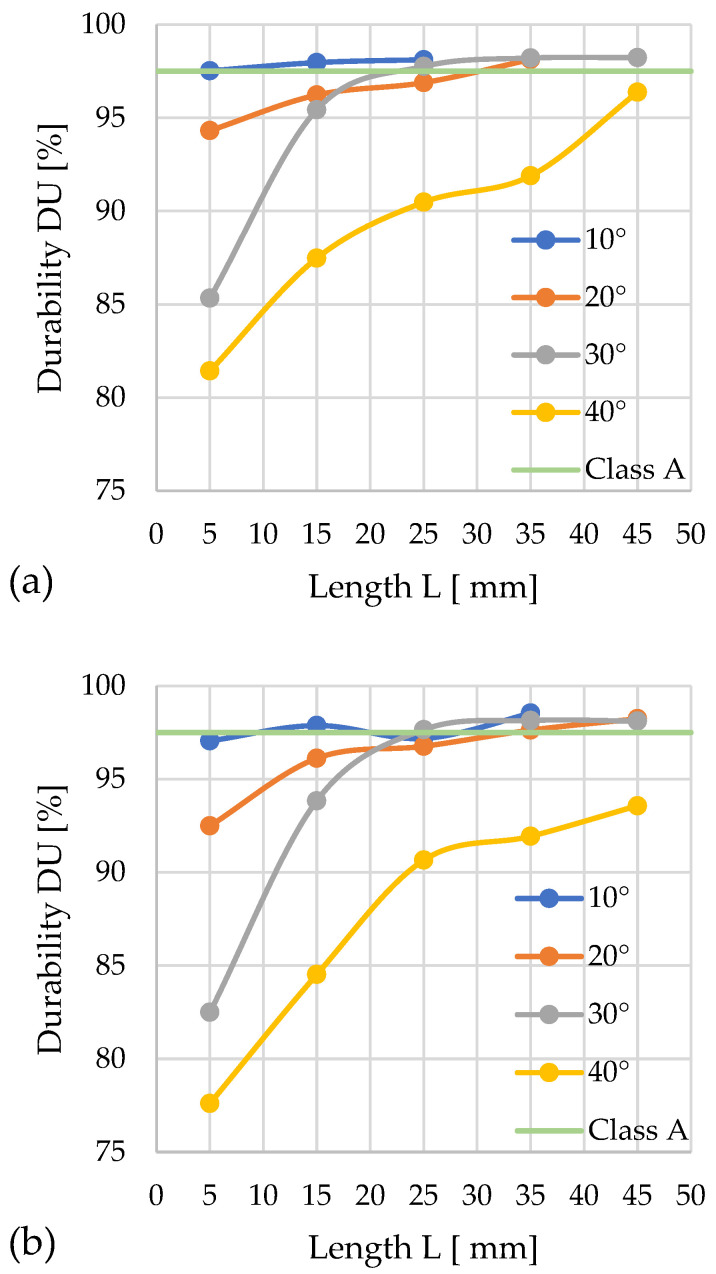
Effects of channel length (*L*), cone angle (*α*), and temperature on pellet durability (*DU*) for sida with 13% moisture: (**a**) size reduction: 12–8 mm; (**b**) size reduction: 10–8 mm.

The graphs showing the change in mechanical durability depending on the geometry of the densification channel are presented in the following figures (Figure 12a,b).

For *D* = 12 mm, the lowest mechanical durability (*DU* = 86.2%) was achieved by pellets compacted with a cone angle of α = 40° and *L* = 5 mm. Among the analyzed configurations, class A was achieved by pellets produced in the following configuration: α = 10° with *L* = 15, 25, 35, 45 mm; α = 20° with *L* = 25, 35, 45 mm; and α = 30° with *L* = 45 mm. The highest *DU* durability was observed for channel lengths of 35 mm, while extending them by 10 mm caused a slight decrease in this value.

For *D* = 10 mm, class A was achieved in the following configuration: α = 10° with *L* = 15, 25, 35, 45 mm and α = 20° with *L* = 35, 45 mm. The highest mechanical durability (*DU* = 98.5%) was obtained for α = 10° and *L* = 35 mm.

Throughout the entire test series, the configuration with the α = 10° angle stood out for its high durability. The other configurations achieved low durability at channel lengths of *L* = 5 and 15 mm. The weakest results were recorded for α = 40°, where no channel length provided the required *DU* value.

An analysis of the relationships between the *DU* parameter change characteristics and the compaction pressure *P* profiles enabled the optimization of the channel geometry for quality class A.

In the case of *D* = 12 mm: α = 10°—*L* = 5 mm and *P* ≈ 237 MPa; α = 20°—*L* ≈ 30 mm and *P* ≈ 687 MPa; α = 30°—*L* = 23 mm and P ≈ 254 MPa; α = 40—none of the configurations met the normative requirements.

In the case of *D* = 10 mm: α = 10°—*L* = 10 mm and *P* ≈ 122 MPa; α = 20°—*L* ≈ 33 mm and *P* ≈ 330 MPa; α = 30°—*L* = 23 mm and *P* ≈ 58 MPa; α = 40°—none of the configurations met the normative requirements.

Among the six possible geometric variants satisfying class A requirements, the configuration characterized by the parameters *D* = 10 mm, α = 30°, and *L* = 23 mm provides the lowest compaction pressure (58 MPa) and minimal energy input for the process.

To facilitate the analysis of the channel geometry’s influence on pellet quality, the data read from the graphs of the influence of *L* on *P*, *DE*, and *DU* for the tested materials and cone angle α were compiled in Table 1. It allows one to read the geometry guaranteeing the achievement of the required *DE* and *DU* values, along with the accompanying process pressure *P*.

Based on the obtained results, it can be concluded that, regardless of the type of material tested, it is easier to achieve the required pellet density (*DE*) than its mechanical durability (*DU*), because achieving *DE* requires the use of a shorter channel *L* than in the case of *DU*. Therefore, it can be stated that *DE* is not a necessary indicator in this type of research, as achieving the required *DU* value always guarantees the achievement of the required *DE* as well. This is the most frequently observed phenomenon, although not always occurring in the case of biomass compacted for energy purposes. Wróbel’s research on the compactibility and densification of lignocellulosic biomass showed that in some cases, biomass during compaction first reaches the required *DU* value, and only later the *DE* value [64]. Examples of biomass where different relationships between density and durability are observed include fir and poplar, when compacted in a dry state. One can also find materials that reach the threshold for the required *DE*, but despite an increase in pressure, do not achieve the corresponding *DU* value [64].

Interestingly, achieving the minimum density equivalent (*DE*) at the minimum pressure occurs at an angle of α = 40° (only for miscanthus and the variant with *D* = 12 mm, this angle is α = 30°). Therefore, for each of the tested materials, the required inlet angle α is similar, and the differences lie only in the channel length *L*.

In the case of an outlet diameter *D* = 12 mm, the pressure value *P* associated with the process is highest for sida (123 MPa) and lowest for silphium (22 MPa). This clearly indicates how much influence the material properties have on the compaction process. Reducing the inlet diameter to 10 mm causes a decrease in pressure. The highest value was recorded for miscanthus (26 MPa) and the lowest, again, for silphium (8 MPa).

In the case of the second pellet quality indicator – *DU* (Durability Index), an angle of α = 40° requires higher pressure values (for miscanthus), while for silphium and sida, it does not allow us to achieve the required *DU* threshold, even at the maximum tested channel length *L*.

According to the data in Table 1, for the *DU*, the lowest pressure accompanies smaller values of the α angle. Depending on the tested material, this angle is different: for miscanthus, α = 10°; for silphium, α = 20°; and for sida, this angle additionally depends on the inlet diameter *D*, being α = 10° for *D* = 12 mm and α = 30° for *D* = 10 mm.

Achieving the *DU* also requires a longer *L*. Miscanthus requires higher compaction pressure values compared to the other materials.

Furthermore, the compaction pressure value is again lower for *D* = 10 mm compared to *D* = 12 mm. This is a clear design guideline for die designers.

Despite minimizing material factors such as moisture content and uniform particle size distribution, the influence of the specific material properties on the *DE*, *DU*, and accompanying process pressure is clearly visible. Even with identical ranges of the densification channel’s geometric variables, the *DE* and *DU* values differ depending on the type of biomass.

The cause of these differences may be the varying structural composition of the studied biomass, i.e., the different proportions of lignin, cellulose, and hemicellulose—where lignin content, being a natural binder, may be particularly significant. This could also be due to differences in stem structure, especially between miscanthus (a monocotyledonous perennial) and sida and silphium (dicotyledonous perennials), which may result in a different course of the densification process. Furthermore, the aforementioned differences can additionally affect the friction coefficient, which in the case of open-chamber densification influences the process course and may determine the length of the die’s cylindrical section.

In regard to the above, analyzing the influence of individual material characteristics and their impact on the process requires further research. Nevertheless, within the scope of this study, it was possible to identify channel geometry variants that enable the production of pellets of appropriate quality for the tested biomass species.

Based on the presented results, it can be concluded that for each material, there is a combination of channel angle and length that allows us to achieve the required density (*DE*) and mechanical durability (*DU*) thresholds.

## 4. Conclusions

Based on the presented test results, the following conclusions can be made:

- The geometry of the compaction channel (*D*, α, and *L*) influences the values of the quality parameters (*DE* and *DU*) of the obtained pellets and the value of the pressure P accompanying the process;
- For the adopted minimum values of *DE* and *DU*, achieving the *DE* threshold requires lower values of *L*, regardless of the material tested;
- The compaction pressure is lower for *D* = 10 mm compared to *D* = 12 mm. In the case of a single channel, this means the throughput of a *D* = 10 mm channel is lower. However, for the entire die, due to the surface fill factor for circles being independent of their diameter, the die’s overall throughput does not change. Therefore, the use of channels with a diameter of *D* = 10 mm is recommended;
- Achieving the *DE* and *DU* thresholds at minimum pressure values requires a channel geometry dedicated to the specific material, as follows: for miscanthus: α = 10°, *L* = 22 mm, *P* = 185 MPa; for silphium: α = 20°, *L* = 26–27 mm, *P* = 42 MPa; and for sida: α = 30°, *L* = 23 mm, *P* = 58 MPa,
- The geometry dedicated to miscanthus (α = 10°, *L* = 22 mm) can be considered a universal geometry. However, if this geometry is used, the compaction process for silphium would be accompanied by a pressure of 47 MPa, while for sida, it would be as high as 450 MPa.
- Further research should be directed towards determining the relationships between the material characteristics of the studied biomass and the required geometry of the die channel, as well as towards investigating the granulation process on pelletizers equipped with dies having hole geometries obtained from the presented studies.

## Figures and Tables

**Table 1:** The die channel geometry enabling the achievement of the required *DE* and *DU* values at the minimal *p* value.

*D* = 12 mm					
Material	α (°)	*DE*: 1 g/cm^3^		*DU*: 97.5%	
		min. *L* (mm)	min. *P* (MPa)	min. *L* (mm)	min. *P* (MPa)
Miscanthus	10	<5	204	13	245
	20	7–8	132	27	276
	30	9	105	39	375
	40	25	190	43–44	357
Silphium	10	<5	57	13	65
	20	<5	29	21	50
	30	9	32	42–43	165
	40	16	22	>45	-
Sida	10	<5	237	5	237
	20	<5	224	30	687
	30	<5	139	23	254
	40	7–8	123	>45	-
***D* = 10 mm**					
Miscanthus	10	<5	121	22	185
	20	<5	51	31–32	211
	30	6–7	37	31–32	200
	40	18–19	26	45	235
Silphium	10	<5	39	15	44
	20	6–7	11	26–27	42
	30	10	11	>45	-
	40	15	8	>45	-
Sida	10	<5	106	10	122
	20	8–9	52	33	330
	30	22	56	23	58
	40	17	17	>45	-

## Data Availability

The original contributions presented in this study are included in the article. Further inquiries can be directed to the corresponding authors.
